# The *Arabidopsis* cytosolic proteome: the metabolic heart of the cell

**DOI:** 10.3389/fpls.2014.00021

**Published:** 2014-02-05

**Authors:** Jun Ito, Harriet T. Parsons, Joshua L. Heazlewood

**Affiliations:** ^1^Joint BioEnergy Institute, Emeryville, CAUSA; ^2^Physical Biosciences Division, Lawrence Berkeley National Laboratory, Berkeley, CAUSA; ^3^Department of Plant and Environmental Sciences, University of Copenhagen, CopenhagenDenmark

**Keywords:** cytosol, ribosome, proteasome, localization, *Arabidopsis*

## Abstract

The plant cytosol is the major intracellular fluid that acts as the medium for inter-organellar crosstalk and where a plethora of important biological reactions take place. These include its involvement in protein synthesis and degradation, stress response signaling, carbon metabolism, biosynthesis of secondary metabolites, and accumulation of enzymes for defense and detoxification. This central role is highlighted by estimates indicating that the majority of eukaryotic proteins are cytosolic. *Arabidopsis thaliana* has been the subject of numerous proteomic studies on its different subcellular compartments. However, a detailed study of enriched cytosolic fractions from *Arabidopsis* cell culture has been performed only recently, with over 1,000 proteins reproducibly identified by mass spectrometry. The number of proteins allocated to the cytosol nearly doubles to 1,802 if a series of targeted proteomic characterizations of complexes is included. Despite this, few groups are currently applying advanced proteomic approaches to this important metabolic space. This review will highlight the current state of the *Arabidopsis* cytosolic proteome since its initial characterization a few years ago.

## INTRODUCTION

**FIGURE 1:**
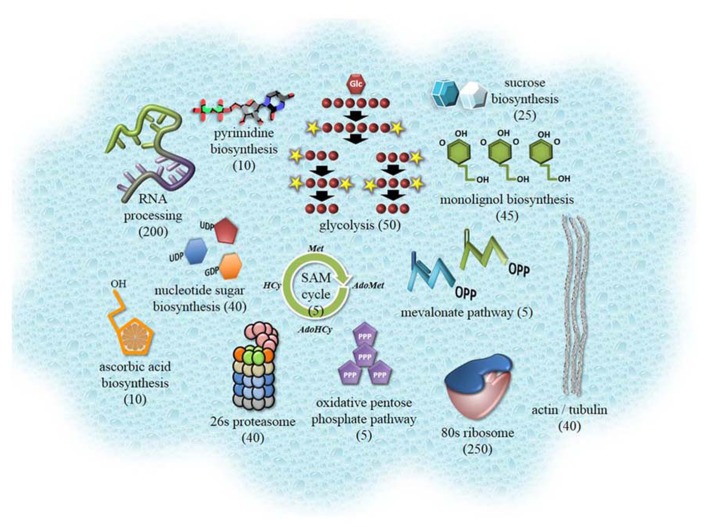
** Cartoon highlighting prominent metabolic processes, proteins, and protein complexes of the plant cytosol.** Components were selected based on the proteomic data outlined in supplementary material of Ito et al. (2010). The number of proteins contributing to each of these components is shown in brackets. This information was obtained from Plant Metabolic Network resource (Zhang et al., 2005) and from MapMan pathway dataset for *Arabidopsis* (Thimm et al., 2004). Collectively, over 2,600 proteins can be assigned to the *Arabidopsis* cytosol when considering proteomics studies, localization with fluorescent proteins and functional curation.

The cytosol is the liquid portion of a cell that contains principle cellular constituents comprising membrane-bound organelles. The cytosol itself lacks membrane compartmentalization. Within its highly concentrated aqueous setting of dissolved ionic solutes, small molecule metabolites and macromolecules, which include nucleic acids and proteins, a wide range of biochemical reactions are known to occur. These include an involvement in glycolysis (Plaxton, 1996), the oxidative branch of the pentose phosphate pathway (Schnarrenberger et al., 1995), protein biosynthesis and degradation (Bailey-Serres et al., 2009; Vierstra, 2009), signal transduction (Lecourieux et al., 2006; Klimecka and Muszynska, 2007), primary and secondary metabolite biosynthesis and transportation (Lundmark et al., 2006; Lunn, 2007; Martinoia et al., 2007; Weber and Fischer, 2007; Krueger et al., 2009), stress response signaling (Yamada and Nishimura, 2008; Cazale et al., 2009; Sugio et al., 2009), and the accumulation of enzymes for defense and detoxification (Laule et al., 2003; Dixon et al., 2009; Sappl et al., 2009). Furthermore, nuclear-encoded organellar proteins are synthesized in the cytosol prior to their import into organelles by targeting peptides (Jarvis, 2008; Prassinos et al., 2008; Huang et al., 2009). Although the cytosol has a multitude of prominent biochemical processes in the eukaryotic cell (**Figure 1**), only two proteome surveys have been carried to date on the plant cytosol. The first study identified 69 abundant proteins in cytosolic samples of soybean root nodules (Oehrle et al., 2008) while the second study identified 1,071 proteins from a large-scale mass spectrometry (MS) analysis of cytosol-enriched fractions from *Arabidopsis thaliana* cell suspension cultures (Ito et al., 2010). Many of the identified proteins were from well-known cytosolic processes (**Figure 1**); although a significant portion of the functionally unclassifiable proteins likely undertake novel roles in the cytosol (Ito et al., 2010). In this review, we will discuss further developments that have occurred from these initial proteomic analyses of the *Arabidopsis* cytosol.

## THE *Arabidopsis* CYTOSOLIC 80S RIBOSOME

The cytosolic ribosome is a major component of the *Arabidopsis* cytosol and has been targeted by a number of studies for analysis by proteomics. A significant proportion of the proteins identified in the cytosolic proteome of *Arabidopsis* are involved in the core biological process of protein biosynthesis and degradation (Book et al., 2010; Ito et al., 2010; Hummel et al., 2012). The ribosome was well-represented amongst these proteins, with 92 previously identified ribosomal protein subunits from 61 of the 80 gene families (Ito et al., 2010). *Arabidopsis* ribosomal proteins have highly conserved sequences that belong to small gene families of two to six members, most of which are expressed (Carroll et al., 2008). A total of 79 of the 80 ribosomal protein families were characterized in purified ribosome preparations from *Arabidopsis* leaves (Giavalisco et al., 2005) and cell suspension cultures (Chang et al., 2005; Carroll et al., 2008). This included the identification of post-translational modifications (PTMs) such as initiator methionine removal, N-terminal acetylation, N-terminal methylation, lysine *N*-methylation, and phosphorylation. These studies represent basic proteomic surveys of the ribosome; more recent analyses have undertaken quantitative approaches to characterize this important protein complex of the cytosol.

Two quantitative proteomic studies have attempted to measure changes in the *Arabidopsis* ribosomal proteome under defined growing conditions. The first quantitative study investigated differential phosphorylation of purified ribosomal proteins from *Arabidopsis* leaves at day and night cycles as a possible mechanism to regulate diurnal protein synthesis (Turkina et al., 2011). Phosphorylation was detected by liquid chromatography (LC)–MS/MS on eight serine residues of six ribosomal proteins: S2-3, S6-1, S6-2, P0-2, P1, and L29-1. Relative quantification of phosphopeptides by differential stable isotope labeling and LC–MS/MS showed significant increases in day to night phosphorylation ratios of ribosomal proteins S6 at Ser-231 (2.2-fold), S6-1 and S6-2 variants at Ser-240 (4.2- and 1.8-fold, respectively), and L29-1 at Ser-58 (1.6-fold). This indicated that differential phosphorylation of these ribosomal proteins are likely mechanisms in modulating diurnal translation in plants (Turkina et al., 2011). The second study performed a label-free absolute quantitative analysis by LC–MS^E^ of immune-purified ribosomal protein paralogs from transgenic *Arabidopsis* leaves in response to sucrose feeding – a treatment known to have a profound effect on plant physiology and gene regulation (Hummel et al., 2012). The extensive families of ribosomal protein paralogs, the ambiguity of their incorporation into ribosomes and the potential alterations to ribosome composition in response to environmental and developmental cues were all factors in carrying out this study. Indeed, out of 204 ribosomal proteins identified by LC–MS/MS, 13 paralogs including S8A, S3aA, L12C, L19A-C, L30B, L8C, L28A, S12A, S12C, L22B, and S7C, as well as the ribosomal scaffold protein RACK1A, showed significant changes in their abundances up to 2.7-fold by LC–MS^E^ in response to sucrose treatments (Hummel et al., 2012). While L28A, L19A, and RACK1 have been shown to be important in normal plant growth and development (Tzafrir et al., 2004; Chen et al., 2006; Yao et al., 2008), the majority display limited phenotypic traits in their mutant plants. Concurrently, multiple ribosomal protein paralogs were shown to be incorporated into ribosomes in both sucrose fed and unfed plants. It was surmised from these results that the *Arabidopsis* cytosolic ribosomes undergo variable alteration to their protein paralog compositions in reaction to changing external conditions (Hummel et al., 2012).

## THE *Arabidopsis* CYTOSOLIC 26S PROTEASOME

The 26S proteasome is a complex of approximately 2.5 MDa which is responsible for the proteolytic degradation of most ubiquitylated proteins. Ubiquitylated protein degradation regulates processes such as the cell cycle, organ morphogenesis, circadian rhythms, and environmental response (Vierstra, 2009). The proteasome consists of a 28-subunit core protease (CP), which houses the active sites for protein and peptide hydrolysis, and a regulatory particle (RP) of at least 18 subunits which regulates substrate recognition, unfolding, and access to the CP. The architecture is highly conserved amongst eukaryotes but recent affinity purification of the 26S complex from *Arabidopsis* has revealed that although the plant 26S proteasome is analogous to that of the human and yeast (Kim et al., 2011), important differences exist.

In *Arabidopsis*, as in other plant groups, almost all subunits in both the CP and RP are encoded by duplicate genes of at least 90% homology, of which few appear to be pseudogenes (Book et al., 2010). Complexes containing all subunit duplicants have been purified from whole plants and characterized by MS (Yang et al., 2004; Book et al., 2010). It is not known yet whether duplicants are inserted into the 26S proteasome randomly or specifically. If these subunit “duplicants” are functionally specific, this raises the possibility of localized regulation of specific protein groups by populations of 26S proteasomes containing specific subunit duplicants/variants. In mutant backgrounds for the RPT2a/b subunit (Lee et al., 2011), complementation studies revealed functional redundancy between duplicants. However, double rpt2a/rpt2b knockout mutants exhibited a more severe phenotype that either single mutant, suggesting redundancy is only partial. RPN2a has uniquely been shown to be unregulated in response to increased sucrose concentrations, implicating a RPN2a-complex in the degradation hexokinase signaling pathway proteins (Sun et al., 2012). Likewise, single RPN5a/b mutants are phenotypically different and double mutants are lethal (Book et al., 2009; Serino and Pick, 2013). Together, these pieces of evidence point toward neofunctionalization of gene duplicants, supporting the idea of multiple populations of complexes within a whole plant.

Most of what is known about the plant 26S proteasome comes from yeast studies and has been reviewed previously (Finley, 2009; Vierstra, 2009). However, a recent study of RPN10 in *Arabidopsis* shows that important functional differences exist, at least in recognition of ubiquitylated substrates (Lin et al., 2011). Further unique properties of the *Arabidopsis* 26S proteasome include a much greater degree of ubiquitylation of subunits than has been observed in yeast (Peng et al., 2003; Book et al., 2010). Subunits became ubiquitylated when still assembled as a complex, implying that this modification performed a function beyond tagging subunits for degradation after complex disassembly. Accessory proteins help assemble the complex and recognize and recruit ubiquitylated substrates. A number of proteins homologous to yeast accessory proteins co-purified with the *Arabidopsis* 26S proteasome, as well as some novel putative accessory proteins not found in yeast (Book et al., 2010). An interesting question for future studies is whether certain accessory proteins associate with particular subunit variants/duplicants.

An important aim in understanding plant 26S proteasome function is to understand the relationship between subunit composition, and specific protein degradation in response to changes in internal and external environments. Given the high identity of many of these subunits, this will involve a significant challenge for characterization by MS. Nonetheless, together with the recent analysis of the ubiquitylated proteome in *Arabidopsis* (Kim et al., 2013), such work will undoubtedly expand our understanding of signaling and process regulation related to this important cytosolic protein complex.

## POST-TRANSLATIONAL MODIFICATIONS

The ability to routinely identify and quantify PTMs represents a grand challenge in the field of proteomics (Heazlewood, 2011). However, few proteomic studies have targeted a subcellular compartment to specifically characterize PTMs (de la Fuente van Bentem et al., 2006; Ito et al., 2009). To the best of our knowledge, no such survey has ever been conducted on highly purified cytosolic fractions from *Arabidopsis*. Aside from the detailed analyses of the purified cytosolic complexes 80S ribosome and 26S proteasome outlined above, PTMs identified on cytosolic localized proteins are largely the result of large-scale PTM-targeted studies. In *Arabidopsis*, this has included phosphorylation (Heazlewood et al., 2008), *N*-linked glycosylation (Zielinska et al., 2012), ubiquitination (Kim et al., 2013), methionine oxidation (Marondedze et al., 2013), *S*-nitrosylation (Fares et al., 2011), and acetylation (Finkemeier et al., 2011). With few exceptions, these studies comprise collections of identified sites and do not generally explore the functional implication of a PTM. However, a number of more detailed investigations have identified the importance of PTMs on proteins localized to the cytosol. Entry into the cytosolic oxidative pentose phosphate pathway (OPPP) is catalyzed by glucose-6-phosphate dehydrogenase (G6PD) which is encoded by AT3G27300 and AT5G40760 in *Arabidopsis*. Large-scale phosphoproteomic studies have identified phosphorylation sites on both cytosolic isoforms. Recently it was demonstrated that the phosphorylation of AT5G40760 at Thr-467 increased G6PD activity fourfold (Dal Santo et al., 2012). Glycolysis represents a key metabolic pathway in the plant cytosol. The sixth step in this pathway is catalyzed by glyceraldehyde-3-phosphate dehydrogenase (GAPDH) and represents the beginning of a net gain in ATP and NADH. In *Arabidopsis*, the step is encoded by a small gene family, a member of which has been identified as lysine acetylated (AT1G13440) in *Arabidopsis*. It was also demonstrated that the acetylation of Lys-130 inhibited the activity of this enzyme *in vitro* and consequently this PTM may represent a regulatory mechanism for this step in the pathway (Finkemeier et al., 2011). GAPDH encoded by AT1G13440 also contains *N*-glycosylation and numerous phosphorylation sites according to a number of targeted PTM studies (Heazlewood et al., 2008; Zielinska et al., 2012). The functional roles, if any, of the many thousands of PTMs on cytosolic localized proteins will likely take many years to accurately characterize. Recently many of these sites were incorporated into the MASCP Gator, the *Arabidopsis* proteomics aggregation portal (Mann et al., 2013). It is envisaged that the inclusion of this information into such a utility will enable the community to better leverage these data for future functional analyses.

## UTILIZATION OF THE *Arabidopsis* CYTOSOLIC PROTEOME

Establishing the subcellular location of a protein is an important factor in determining its function (Chou and Cai, 2003). MS analysis of purified organelles or cellular compartments and chimeric fluorescent fusion proteins are two common experimental methods used to define subcellular localizations of *Arabidopsis* proteins (Heazlewood et al., 2007; Tanz et al., 2013). Over 2,200 proteins contain information indicating a cytosolic localization in *Arabidopsis* (**Table 1**), which comprises nearly 25% of all experimentally localized proteins in the SUBcellular Arabidopsis database (SUBA). A large proportion of these cytosolic proteins have been identified in multiple subcellular compartments, especially in the case of proteomic approaches. It is therefore ideal, though often not the case, that protein localization is confirmed using complementary methods (Millar et al., 2009).

**Table 1:** A survey of cytosolic proteins experimentally localized in *Arabidopsis* from the SUBA database as of November 2013 (Tanz et al., 2013).

	MS/MS	FP	Total	Overlap
All locations	7891	2647	9319	1219
Cytosol	1808	580	2262	126

*MS/MS indicates proteins identified through subcellular proteomics studies; FP are proteins localized using a fluorescent protein tag. The overlap between FP and MS/MS for cytosolic proteins is significantly worse than all proteins localized in the SUBA database. Possibly reflecting poor attention to this subcellular space and its processes by the research community.*

Several recent reports have used data from the *Arabidopsis* cytosolic proteome to confirm functional interpretations supporting a localization in the cytosol. Overall, they exemplify the practicality of this subcellular proteome for verifying the cytosolic localizations of different proteins. Glyoxylate reductase (GLYR) is a central enzyme in the γ-aminobutyrate (GABA) metabolic pathway, where it catalyzes the detoxification of glyoxylate and succinic semialdehdye (Ching et al., 2012). The two plant isoforms GLYR1 and GLYR2 were believed to localize to the cytosol or peroxisomes, and plastid, respectively. Conflicting reports of *Arabidopsis* GLYR1 (At3g25530) localizing in the cytosol (Simpson et al., 2008) or the peroxisome (Reumann et al., 2009) had implications for defining its exact metabolic roles and the compartmentation of the GABA and photorespiratory pathways. This was resolved by visualizing N-terminal green fluorescent protein (GFP)-tagged GLYR1 in *Arabidopsis* suspension-cultured cells, leaves and seedlings and tobacco BY-2 suspension-cultured cells, where it was observed to exclusively localize in the cytosol (Ching et al., 2012). Its identification by MS as a major protein in the cytosolic proteome of *Arabidopsis* cell suspensions was cited as further evidence of this finding (Ito et al., 2010; Ching et al., 2012).

The *Arabidopsis* translation elongation factor eEF-1Bβ1 (EF1Bβ, At1g30230) is involved in plant cell wall biosynthesis and it is essential for normal plant development (Hossain et al., 2012). *Arabidopsis* plants with T-DNA insertions in their EF1Bβ gene display a dwarf phenotype, with alterations to their vascular morphology and inflorescence stem structures and 38 and 20% reductions in total lignin and crystalline cellulose content, respectively. By transforming *Arabidopsis* plants with a 35S promoter-controlled EF1Bβ fused with yellow fluorescent protein (EF1Bβ-YFP), the subcellular locations of EF1Bβ were visualized in the plasma membrane and cytosol (Hossain et al., 2012). These observations agreed with MS analyses of the *Arabidopsis* plasma membrane (Mitra et al., 2009) and cytosol proteomes (Ito et al., 2010), with EF1Bβ identified in both subcellular compartments.

An evolutionary and structural analysis of a human disrupted in schizophrenia 1 (DISC1) protein conducted orthology searches of non-vertebrate reference organisms such as *Dictyostelium*, *Trichoplax*, *Monosiga*, and *Arabidopsis* (Sanchez-Pulido and Ponting, 2011). This study found that while most DISC1 orthologs lacked any experimental evidence of their functions, the *Arabidopsis* DISC1 ortholog (At5g25070) is ubiquitously expressed in various tissues and developmental stages and is a constituent of the *Arabidopsis* cytosolic proteome (Ito et al., 2010). This was strikingly similar to human DISC1, which is expressed in a wide range of tissues and also cytosol-localized (Sanchez-Pulido and Ponting, 2011).

## EXPANDING THE *Arabidopsis* CYTOSOLIC PROTEOME

A computational analysis of the *Arabidopsis* proteome estimated that the cytosolic proteome may contain around 5,400 ± 650 proteins (Ito et al., 2010). This indicates that the current experimental set of 2,262 proteins likely represents only about 40% of the cytosolic proteome (Table 1). A dissection of fluorescent protein-based localization studies of *Arabidopsis* proteins (Table 1) reveals that many members were also identified in the *Arabidopsis* cytosolic proteome (recent examples include Ching et al., 2012; Christ et al., 2012; Hossain et al., 2012; Li et al., 2012; Lu et al., 2012; McLoughlin et al., 2012; Witz et al., 2012). However, there are many examples of FP-tagged proteins that have been localized to the cytosol and not identified by proteomic surveys (some recent studies include Gaber et al., 2012; Hernandez et al., 2012; Kwon et al., 2012; Lu et al., 2012; McLoughlin et al., 2012; Rautengarten et al., 2012; Vadassery et al., 2012; Witz et al., 2012). The inclusion of complementary subcellular datasets such as those available from the gene ontology database AmiGO (Carbon et al., 2009) and UniProtKB (Magraneand and UniProt Consortium, 2011) can also be used to capture some of these missing cytosolic proteins. Nearly 2000 *Arabidopsis* proteins are designated as cytosolic by AmiGO, while about 1,300 *Arabidopsis* proteins are allocated to the cytosol by the UniProt Protein Knowledgebase. Incorporating these data with the proteomic and fluorescent protein information, the total number of *Arabidopsis* proteins with some cytosolic designation is 2604 distinct members or about 50% of the computationally derived proteome. It should be noted that the “experimental” figure of ca. 2,600 does not account for false positives resulting from proteins with multiple subcellular designations. Over 1,400 of these proteins also have non-cytosolic assignments by either MS or fluorescent protein localizations according to SUBA (Tanz et al., 2013).

While proteomics has identified a considerable proportion of the computationally derived cytosolic proteome (around 30%), the shortfall can be readily explained and include: many proteins are not abundant and thus not easily detected by MS, many proteins could be expressed in tissue(s) other than cell suspension cultures or only under certain conditions (i.e., at a specific stage of plant development or in response to stress) and most significantly only one out of the nearly 120 proteomic analyses of various subcellular compartments from *Arabidopsis* has been performed on its cytosolic fraction (Heazlewood et al., 2007; Ito et al., 2010). In contrast, studies in *Arabidopsis* in the areas of respiration and photosynthesis have benefited tremendously from the characterization of their proteomes across different organs and tissues, developmental stages, and growth conditions (Lee et al., 2008; van Wijk and Baginsky, 2011). In order to better understand its dynamics, future analyses of the *Arabidopsis* cytosolic proteome will also need to reach this level of diversity.

A critical factor in performing in-depth proteomic analysis of the cytosol from plants will be to obtain relatively pure cytosolic fractions from this material. Isolating the cytosolic fraction from *Arabidopsis* cell suspensions relies on enzymatic generation of protoplasts and their disruption by gentle pressure to maintain organelle integrity, followed by organelle removal by differential centrifugation (Ito et al., 2010). Unlike uniform heterotrophic cell suspensions, cytosol purification from plants requires extra steps including the removal of chloroplasts. A study of protein localization between cytosol and chloroplasts of *Arabidopsis* seedlings developed a method for isolating the cytosolic fraction from protoplasts of seedlings (Estavillo et al., 2011). The addition of density centrifugation was necessary to remove broken protoplasts and intact chloroplasts, respectively, from the seedling cytosolic fraction (Estavillo et al., 2011, 2014). Employing immunoblotting or MS-based quantitation against subcellular markers to assess organelle contamination during the extraction process (Ito et al., 2010), this method could be further refined to generate high-purity cytosolic fractions from many types of *Arabidopsis* plant material for proteomic analysis.

Sub-fractionation of the cytosol is an effective way to reduce its protein complexity and to improve MS/MS identification of low abundant cytosolic proteins. Unlike mitochondria and plastids, the cytosol lacks defined membrane-bound compartments that can be further sub fractionated (Eubel et al., 2007; Ferro et al., 2010). However, isolating soluble protein complexes from the *Arabidopsis* cytosol has been shown to be relatively straight forward. As outlined above, both the 80S ribosome and the 26S proteasome have been isolated and extensively characterized by MS (Yang et al., 2004; Chang et al., 2005; Giavalisco et al., 2005; Carroll et al., 2008; Book et al., 2010; Turkina et al., 2011; Hummel et al., 2012). Beyond these examples, sub-fractionation of other cytosolic protein groups will likely rely on affinity purification techniques tailored to the physiochemical properties of target proteins to simplify complex mixtures and enrich for low abundant proteins. In non-plant systems approaches have included immobilized heparin chromatography to fractionate cytosolic proteins from human breast cancer MCF-7 cells (Shefcheck et al., 2003). Approximately 300 low-abundant cytosolic proteins were detected by two-dimensional gel electrophoresis (2-DE) of heparin fractions, and they were not present on 2-DE separations of total cytosolic protein mixtures (Shefcheck et al., 2003). Finally, LC–MS/MS analysis of tandem biomimetic affinity pre-fractionation of rat liver cytosol proteins identified 665 unique rat proteins, which was significantly more than the 371 proteins in the unfractionated cytosol (Tan et al., 2009).

## PERSPECTIVES

There is tremendous scope to extend our current knowledge of the multitude of reactions that take place in the plant cytosol. Few studies have employed quantitative proteomic approaches to study cytosolic components revealing a lack of attention to this important compartment. Similarly, the characterization and analysis of PTMs of cytosolic proteins will be a significant challenge in the future. Recent reports of cytosolic localizations of *Arabidopsis* proteins by fluorescent protein tagging showed that while a number of them were identified in the cytosolic proteome, many others were not. Future comparative analysis of cytosolic proteomes of different plant tissues grown under various environmental conditions is essential to better understand its dynamics and to unravel its complexity. Isolating pure cytosolic fractions and their sub-fractions from diverse sources of plant material for LC–MS/MS analysis will be key factors to achieve this aim.

## AUTHOR CONTRIBUTIONS

The manuscript was devised by Jun Ito and written by Jun Ito, Harriet T. Parsons, and Joshua L. Heazlewood. Figure and Table were constructed by Joshua L. Heazlewood.

## Conflict of Interest Statement

The authors declare that the research was conducted in the absence of any commercial or financial relationships that could be construed as a potential conflict of interest.
